# A method to identify, dissect and stain equine neuromuscular junctions for morphological analysis

**DOI:** 10.1111/joa.13747

**Published:** 2022-09-10

**Authors:** Stephen D. Cahalan, Justin D. Perkins, Ines Boehm, Ross A. Jones, Thomas H. Gillingwater, Richard J. Piercy

**Affiliations:** ^1^ Comparative Neuromuscular Diseases Laboratory, Department of Clinical Science and Services Royal Veterinary College London UK; ^2^ Edinburgh Medical School: Biomedical Sciences University of Edinburgh Edinburgh UK; ^3^ Euan MacDonald Centre for Motor Neuron Disease Research University of Edinburgh Edinburgh UK; ^4^ Biozentrum University of Basel Basel Switzerland

**Keywords:** comparative anatomy, equine, large mammal, muscle fibre teasing, neuromuscular junction, NMJ‐morph, synapse

## Abstract

Morphological study of the neuromuscular junction (NMJ), a specialised peripheral synapse formed between a lower motor neuron and skeletal muscle fibre, has significantly contributed to the understanding of synaptic biology and neuromuscular disease pathogenesis. Rodent NMJs are readily accessible, and research into conditions such as amyotrophic lateral sclerosis (ALS), Charcot–Marie–Tooth disease (CMT), and spinal muscular atrophy (SMA) has relied heavily on experimental work in these small mammals. However, given that nerve length dependency is an important feature of many peripheral neuropathies, these rodent models have clear shortcomings; large animal models might be preferable, but their size presents novel anatomical challenges. Overcoming these constraints to study the NMJ morphology of large mammalian distal limb muscles is of prime importance to increase cross‐species translational neuromuscular research potential, particularly in the study of long motor units. In the past, NMJ phenotype analysis of large muscle bodies within the equine distal pelvic limb, such as the *tibialis cranialis*, or within muscles of high fibrous content, such as the *soleus*, has posed a distinct experimental hurdle. We optimised a technique for NMJ location and dissection from equine pelvic limb muscles. Using a quantification method validated in smaller species, we demonstrate their morphology and show that equine NMJs can be reliably dissected, stained and analysed. We reveal that the NMJs within the equine *soleus* have distinctly different morphologies when compared to the *extensor digitorum longus* and *tibialis cranialis* muscles. Overall, we demonstrate that equine distal pelvic limb muscles can be regionally dissected, with samples whole‐mounted and their innervation patterns visualised. These methods will allow the localisation and analysis of neuromuscular junctions within the muscle bodies of large mammals to identify neuroanatomical and neuropathological features.

## Graphical abstract

This equine NMJ method paper tackles the staining and analysis of large mammalian muscles. Equine NMJ morphology is revealed to be similar to other vertebrates. Clear differences exist between the equine soleus and other equine pelvic limb muscle NMJs.

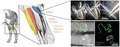

## 1 INTRODUCTION

The neuromuscular junction (NMJ) is a specialised, excitatory, chemical synapse formed between a lower motor neuron and a skeletal muscle fibre. The NMJ connects a pre‐synaptic axonal terminal to a post‐synaptic region rich in acetylcholine receptors (AChR), allowing for neuronal activation of muscle contraction (Rana et al., 2017). As NMJs are relatively large and readily accessible, they have been a mainstay of vertebrate (Tokuyama et al., 2018; Bermedo‐García et al., 2018; Desaki & Uehara, 1981; Heuser & Reese, 1973) and invertebrate (Keshishian et al., 1996) neuromuscular research from the 1800s (Kuhne, 1887) until the present day; rodent studies, in particular, continue to reveal the cellular changes involved in synapse formation (Roche et al., 2014), degeneration (Gillingwater & Ribchester, 2001) and repair (Lawrence et al., 2008).

In contrast to the relative abundance of small mammalian NMJ research, there is a paucity of corresponding large mammalian data, and in humans, this work is restricted by ethical and logistical issues (Boehm, Miller, et al., 2020; Jones et al., 2017). Common to NMJ quantification across species, large muscles that cannot be prepared as wholemounts for microscopic examination have generally required cryosectioning prior to immunolabelling of NMJs, thereby generating sectioning and refraction‐related imaging artefacts, which increase analytical subjectivity and limit possible analyses. Small rodent models present an obvious advantage in this regard: their muscles are more readily dissected and whole‐mounted, and their innervation patterns are more easily identified along with fewer sectioning‐related artefacts (McArdle et al., 1981; Murray et al., 2010; Sleigh, Burgess, et al., 2014). This allows for more accurate quantification of NMJ development (Mech et al., 2020), degeneration (Sleigh, Grice, et al., 2014) and repair (Martineau et al., 2018). The caveats inherent in using (the much smaller) rodents to model human NMJ pathobiology (these issues are expanded on in Cahalan et al., 2022) create a need for large mammal NMJ analysis (Pereira et al., 2020; Sullivan et al., 2020).

Recent comparative mammalian pelvic limb NMJ data have revealed marked morphological heterogeneity and suggest that large animal neuromuscular models have translational benefits in widening our anatomical and pathophysiological understanding (Boehm, Alhindi, et al., 2020). We and others have shown that many properties of nerve conduction velocity and muscle physiology––shortening velocities, metabolic and enzymatic activities––are similar in horses to those of other mammals, including humans (Cercone et al., 2019; Draper & Piercy, 2018; Rivero & Hill, 2016), so we hypothesised that basic similarities would also be present in equine NMJ morphology. To investigate further, we needed to adapt existing muscle fibre dissection and processing techniques (Boehm, Alhindi, et al., 2020) to equine pelvic limb muscles, selected for direct comparison with this dataset: the *tibialis cranialis*, *extensor digitorum longus* and the *soleus*.

Detailed descriptions of muscle fibre dissections date from the early 1900s when Huber isolated individual rabbit muscle fibres using a 75% hydrochloric acid solution (Huber, 1916). Since then, both physical (Matsuoka et al., 1992) and chemical (Loeb et al., 1987) muscle fibre separation methods have been used, generally to quantify muscle fibre length (Huber, 1916; Loeb et al., 1987; Matsuoka et al., 1992). Over a similar period, acetylcholinesterase (AChE) staining techniques were developed (for review see Couteaux, 1998), with a particular breakthrough in 1949 when AChE was first used as a substrate in light microscopy (Koelle & Friedenwald, 1949), and since then modified to investigate NMJ distribution across species (Karnovsky & Roots, 1964; Meyers & Hermanson, 1994; Ypey, 1978), including in the horse (Cheetham et al., 2008; Reesink et al., 2013). Teased muscle fibre work in horses has been limited to two studies quantifying muscle fibre length in elbow extensor muscles (Ryan et al., 1992) and the *sternocephalicus* muscle (Zenker et al., 1990). To our knowledge, there are no peer‐reviewed data describing the morphological structure of equine NMJs.

Here, we describe a technique for processing equine pelvic limb muscles for NMJ analysis, identifying gross NMJ distribution, dissecting and teasing muscle fibres, and using immunofluorescent staining and microscopy to illustrate the neuromuscular phenotype. This technique can easily be applied to other large muscles to perform detailed neuromuscular analysis in horses and other large mammals.

## 2 MATERIALS AND METHODS

### 2.1 Animals

5 Welsh Mountain ponies (3 female, 2 neutered males) were used for all experiments (Table 1). Animals were kept outside at grass pasture with ad lib access to water. Euthanasia was conducted (for reasons unrelated to this study) via intravenous barbiturate overdose. All muscles were dissected immediately post mortem. Muscle samples (Table 2) were obtained with ethical approval of the Royal Veterinary College's Clinical Research Ethical Review Board.

**TABLE 1:** List of ponies sampled, including age, weight, sex, height and breed. *N* = neutered

Subject	Age (years)	Mass (kg)	Sex	Height (cm)	Breed
Pony 1	4.5	180	Female	110	Welsh Mountain Section A
Pony 2	7.5	230	Male N	120	Welsh Mountain Section A
Pony 3	5	400	Female	140	Welsh Mountain Section D
Pony 4	8	350	Male N	125	Welsh Mountain Section A
Pony 5	8	350	Female	125	Welsh Mountain Section A

**TABLE 2:** List of muscles sampled, including abbreviation, origin, insertion, action, mean and reported mean percentage fast twitch muscle fibre data, with references

Muscle	Abbreviation	Origin	Insertion	Action	Mean % fast twitch (SD)	Reported mean % fast twitch (SD)	Reference
Tibialis cranialis	TC	Tibial lateral condyle and crest	Cuneiforms and metatarsal III	Flexion of hock (Tarsus)	58.5% (±14.46)	64.6% (±15.6)	Valberg et al. (2015)
Extensor digitorum longus	EDL	Femoral extensor fossa	Distal phalangeal extensor process	Extension of digits and flexion of hock (Tarsus)	75% (±9.69)	89.4% (±13.4)	Kawai et al. (2009)
Soleus	SOL	Fibular head	Gastrocnemius aponeurosis	Minimal hock (Tarsus) extension, many muscle spindles ‐proprioceptive function?	<1%	1%–14% (±15)	Meyers and Hermanson (2006) Kawai et al. (2009)

### 2.2 Reagents

The reagents and quantities/concentrations used are listed in Table 3.

**TABLE 3:** Details of the reagents used in NMJ location, NMJ immunofluorescence and wholemount muscle fibre mounting

Item	Manufacturer	Description/catalogue no.	Dose/concentration
Acetylcholinesterase Staining			
0.1 M Tris buffered saline (TBS)	Merck Sigma‐Aldrich	P4417‐100TAB	3 L
Sodium phosphate dibasic (Na2HPO4)	Merck Sigma‐Aldrich	255,793	22.56 g (0.158 M)
Sodium phosphate monobasic (NaH2PO4)	Merck Sigma‐Aldrich	S3139	70.5 g (0.58 M)
Acetylthiocholine iodide	Merck Sigma‐Aldrich	A5626	4.34 g
Glycine	Merck Sigma‐Aldrich	G7126	11.26 g
Copper (II) sulphate pentahydrate (CuSO4)	Merck Sigma‐Aldrich	C2284	6.19 g
Fixation and immunolabelling			
4% Paraformaldehyde in TBS	Merck Sigma‐Aldrich	P6148	Made fresh or stored for no more than 12 h prior at 4°C
Triton X‐100	Merck Sigma‐Aldrich	T8787	2% Triton X‐100 in TBS (Blocking)
Bovine Serum Albumin (BSA)	Merck Sigma‐Aldrich	A9647	4% solution in TBS
α‐Bungarotoxin conjugated to Alexa Fluor 594	Invitrogen Thermofisher Scientific	B13423	Used at 1:500 in TBS
Anti‐Neurofilament‐associated antibody	Developmental Studies Hybridoma Bank (DSHB)	Mouse anti‐3A10 AB_531874	Used at 1:50 in BSA/TBS
Anti‐Synaptic vesicle protein 2	Developmental Studies Hybridoma Bank (DSHB)	Mouse anti‐SV2 AB_2315387	Used at 1:50 in BSA/TBS
Antimouse IgG Mouse IgG (H + L) highly cross‐adsorbed secondary antibody	Invitrogen Thermofisher Scientific	A‐11029	Used at 1:200, diluted in TBS.
Wholemount muscle fibre mounting			
Mowiol® 4–88	Merck Sigma‐Aldrich	81,381	2.4 g
0.2 M Tris‐Cl	Merck Sigma‐Aldrich	75,837	12 ml pH 8.5
Glycerol	Merck Sigma‐Aldrich	G9012	6 g
1,4‐Diazabicyclooctane DABCO 2.5%	Merck Sigma‐Aldrich	D27802	2.5% w/v

### 2.3 Equipment and software

The equipment used is listed in Table 4. ImageJ version 2.1.0/1.53c (macOS v12.3.1) (http://rsb.info.nih.gov/ij/) was used for projecting Z‐stack images and combined with the aNMJ‐morph workflow (Jones et al., 2016; Minty et al., 2020) to quantify NMJ morphological variables.

**TABLE 4:** Details of the equipment used in muscle fibre dissection and teasing, NMJ immunofluorescence and wholemount muscle fibre mounting and imaging

Item	Manufacturer	Description
Dissection scope	Zeiss	Zeiss Stemi DV4 Stereo Microscope 8×–32×
SuperFine Vannas Scissors	WPI	8 cm long, 3 mm blades, 0.015 mm × 0.015 mm tips
Curved Iris Forceps	WPI	Full Curve #4
Dumont Mini Forceps	Fine Science Tools	Item No. 11200–10 ‐ Style #3
Dumont Fine Forceps	Fine Science Tools	Item No 11254–20 Straight/Inox/11 cm
SYLGARD 184	Dow Corning	1.1KG—Silicone Elastomer, Flowable,
90 mm Petri Dishes	Thermo Scientific™	Sterilin™ Standard
Minutiens insect pins	Austerlitz 0.20	~ 0.2 mm
Rocker	VWR	444–0116
2 ml Eppendorf tubes	Eppendorf	3810X
15 ml and 50 ml Falcon tubes	BD Falcon	352,097 and 352,070
Magnetic stirrer and stir bar	VWR	442–0883 and 442–0272
12‐well tissue culture plates	BD Falcon	353,047
Magnetic stirrer and stir bar	VWR	442–0883 and 442–0272
50 ml conical flask	Corning	70,980
Superfrost™ Ultra Plus Adhesion Slides	Thermofisher Scientific	J3800AMNT
Rectangular coverslips	Thermofisher Scientific	22X30‐1
Leica ASP8 inverted scanning confocal microscope	Leica	SP8

### 2.4 Acetylcholinesterase (AChE) staining

**FIGURE 1:**
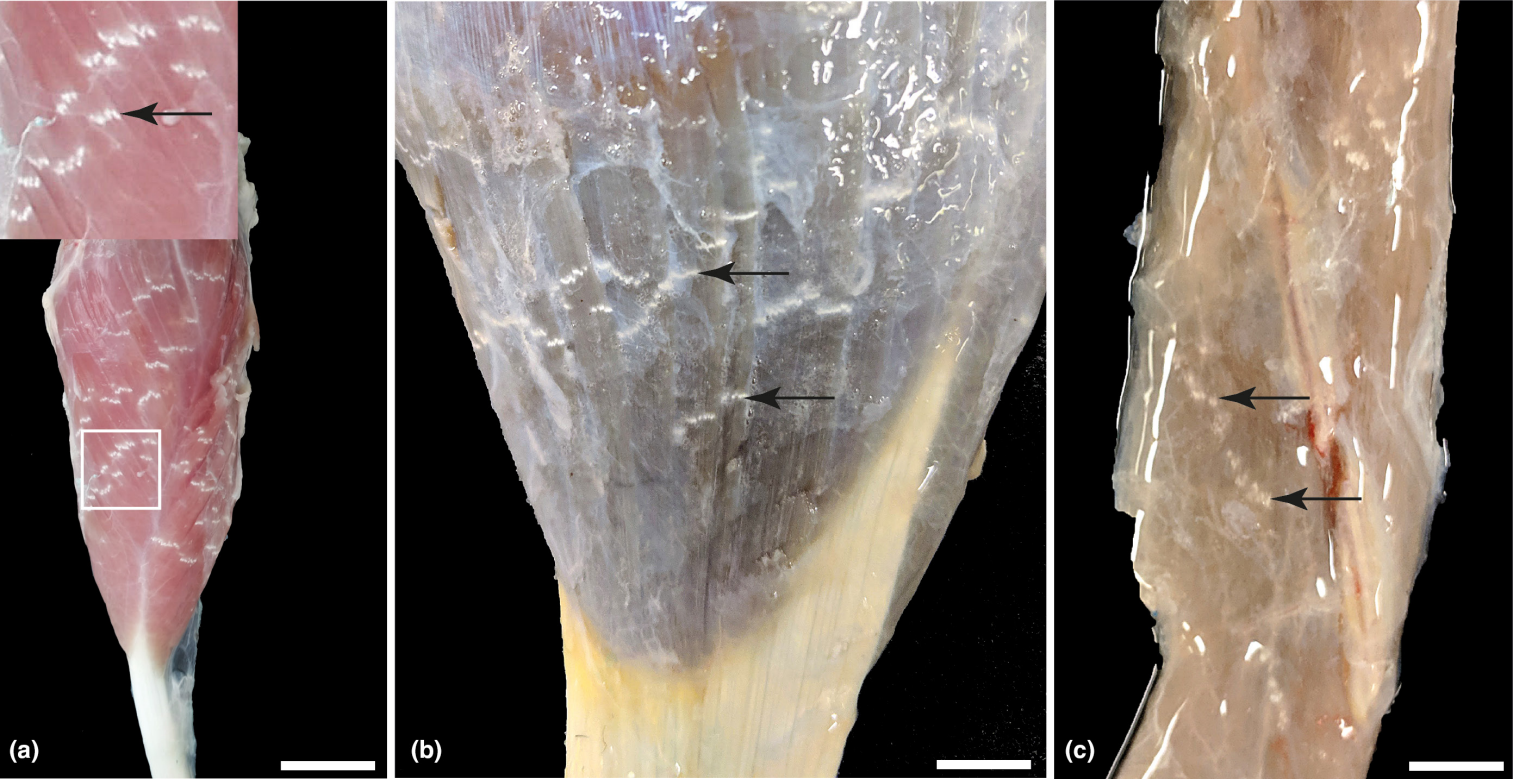
Acetylcholinesterase staining of endplate distribution within *cranialis tibialis*, *extensor digitorum longus* and *soleus* muscles: (a) caudolateral aspect of dissected *cranialis tibialis* muscle, showing overlapping, white, dotted bands of endplates (arrows), repeating along the entire length of the muscle–inset: Boxed area is shown at a higher magnification, scale bar = 5 cm (b) lateral and distal aspect of *extensor digitorum longus* muscle (c) lateral and distal aspect of *soleus* muscle, scale bars = 1 cm.

AChE staining, adapted from (Cheetham et al., 2008; Couteaux, 1998; Karnovsky & Roots, 1964), was used to visualise the macroscopic endplate distribution in pelvic limb muscles (Figure 1) so that NMJ‐rich areas could be targeted for subsequent sampling and microscopy. 3 L of 0.1 M Tris‐buffered saline solution was made with distilled water, 22.56 g sodium phosphate dibasic and 70.5 g sodium phosphate monobasic and adjusted to pH 6.4. 4.34 g of acetylthiocholine iodide and 11.26 g of glycine were added to the pH‐adjusted TBS solution in a fume hood. Finally, 6.19 g copper (II) sulphate pentahydrate was added while continuously mixing, over the course of 30 min, approximately 0.5–1 g at a time, to avoid precipitation. The mixture was stored at 4°C––and used within 24–48 h. Prior to immersion, muscles were carefully stripped of overlying connective tissue and fat without damaging the perimysium. Then, muscles were submerged for 12 h in the mixture and removed for imaging.

### 2.5 Teasing and staining reagent setup

Sylguard 184 silicone elastomer was prepared by combining the elastomer base with the curing agent at a 10:1 ratio, pouring it into 90 mm Petri dishes, and then allowing it to set for at least 48 h. 16% paraformaldehyde (PFA) in phosphate‐buffered saline (PBS) was diluted in PBS to a 4% working solution and used on the day of preparation. Permeabilisation, blocking, and antibody solutions were all made on the day of preparation (see section 2.7). Mowiol mounting medium was made by adding 2.4 g Mowiol 4–88 and 6 g glycerol to 6 ml distilled water in a 50 ml conical flask, mixing slowly overnight at room temperature on a magnetic stirrer. The following day, prewarmed––to 50°C in a water bath––12 ml 0.2 M Trizma hydrochloride (pH 8.5) was added, and the medium was heated in a water bath at 50°C for 1 h with regular mixing every 10 min. The medium was left to settle for 30 min at room temperature, the supernatant decanted into a graduated cylinder, and volume noted. Finally, 1,4‐diazobicyclo‐[2.2.2]‐octane (DABCO) at 2.5 g per 100 ml was added to reduce fading. The medium was aliquoted into 2 ml Eppendorf tubes to avoid repeated freeze–thaw cycles and stored at −20°C for up to a year.

### 2.6 NMJ immunolabelling—muscle dissection and fibre teasing

**FIGURE 2:**
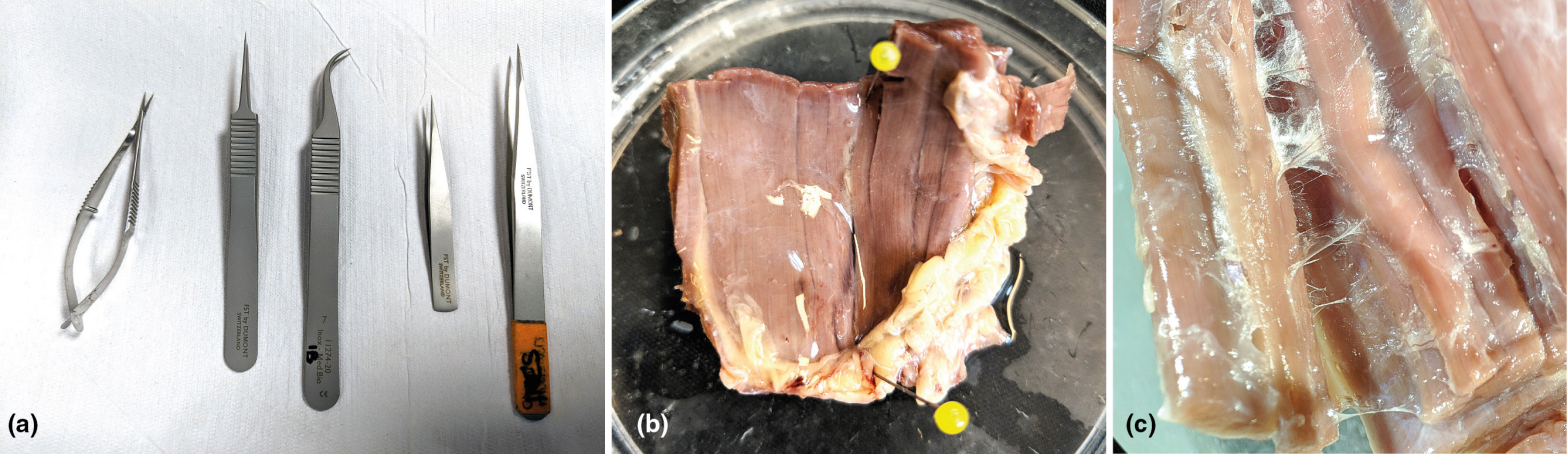
Equipment and setup prior to teasing muscle fibres‐ (a) teasing instruments used, for further reagent and instrument details see Tables 3 and 4. (b) *Tibialis cranialis* muscle pinned to petri dish with Sylgard (TM) base. (c) Illustrating fascial connective tissue in pinned muscle, useful to identify and separate muscle fibre bundles while dissecting.

**FIGURE 3:**
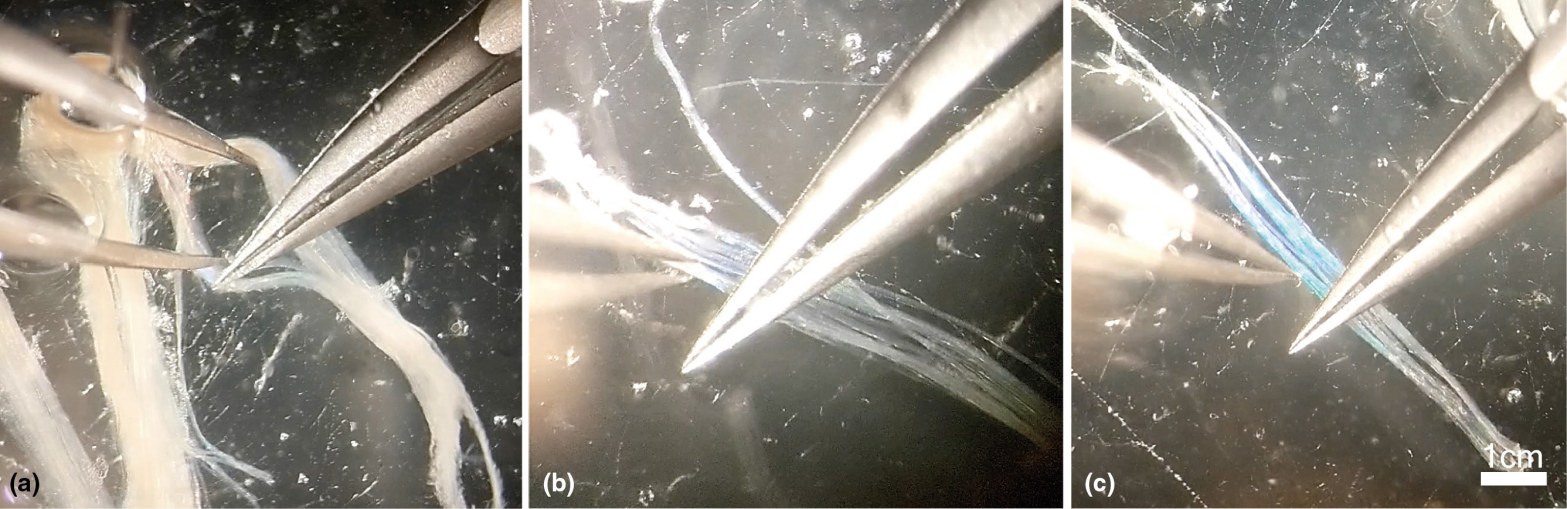
Technique used to tease muscles (a) illustrating use of straight forceps to split muscle fibre bundles, with curved‐bottom forceps for pressing down or holding groups of muscle fibre bundles in place. (b) a size 8 forceps is used to prise apart individual smaller bundles and remove fat and connective tissue. (c) Illustrating the blue‐green iridescent appearance of a cluster of well‐teased muscle fibres.

For whole‐mount muscle preparation of NMJ immunostaining, whole muscles were freshly dissected within 30 min post‐euthanasia and divided into sections of, at a maximum, 4cm^3^ sized pieces and stored in 10 times the muscle volume of 4% PFA at 4°C for a maximum of 12 h. Samples were always chosen from each muscle's same distal superficial location, highlighted in Figure 1. Fixed muscles were then washed three times in TBS for 20 min on a rotator at room temperature, prior to long‐term storage in 0.1 M Sodium Azide in TBS at 4°C. This allowed for repeated muscle fibre teasing and staining for up to a year. Upon dissection, muscles were pinned in a Sylguard silicone elastomer‐covered petri dish using insect pins and immersed in TBS. A dissection microscope was used for all subsequent steps. Individual muscle bundles were identified, connective tissue was detached from between the muscle fibres, and individual fibre bundles were stripped away and carefully teased apart to facilitate antibody penetration, see Figures 2 and 3. See supplemental video for added instruction https://figshare.com/s/40926670e1fc65e038c1. Unwanted material such as fibro‐vascular connective tissue and fat were identified and removed to minimise tissue autofluorescence.

### 2.7 NMJ immunolabelling and mounting

Unless otherwise specified, all permeabilisation, incubation, and washing steps were performed on a rocker at room temperature. Solutions were changed by filling alternate wells of a 12‐well‐plate, then transferring muscle fibres across, rather than replacing the solutions with fibres in situ, to minimise fibre losses. Each well contained between 40–50 teased fibres typically submerged in a volume of 500 μl. Teased fibres were transferred to a 12‐well plate and washed with 1 ml of 0.1 M glycine in TBS pH 10.4 for 1 h. A 10 min TBS wash was followed by a 30 min incubation with Alexa Fluor 594 α‐bungarotoxin (αBTX 1/1000 in TBS). Fibres were permeabilised with 4% Triton X‐100 in TBS for 90 min and then blocked in 4% BSA and 2% Triton X‐100 in TBS solution for 30 min; samples were then incubated for 72 h at 4°C in this same blocking solution containing primary antibodies—mouse neurofilament‐associated (3A10, 1/50) and mouse pan anti‐synaptic vesicle 2 (SV2, 1/50)–labelling axons and nerve terminals, respectively. Samples were then washed four times for 20 min each in TBS. Alexa Fluor 488 goat anti‐mouse IgG secondary (1/400 in TBS) was incubated overnight at 4°C. Samples were washed four more times for 20 min each in TBS. Approximately 0.25 ml of Mowiol mounting medium was dropped onto a glass slide and a bundle of 5–10 fibres, depending on length and diameter were placed within the medium. Using forceps, fibres were spread out and straightened to avoid overlap and curling. Fibres were cover‐slipped and placed flat at 4°C for 24 h. Slides were then stored at −20°C for up to 2 years, without detectable loss of fluorescence whilst avoiding repeated freeze–thaw cycles.

### 2.8 NMJ immunostaining assessment, image acquisition and analysis

**FIGURE 4:**
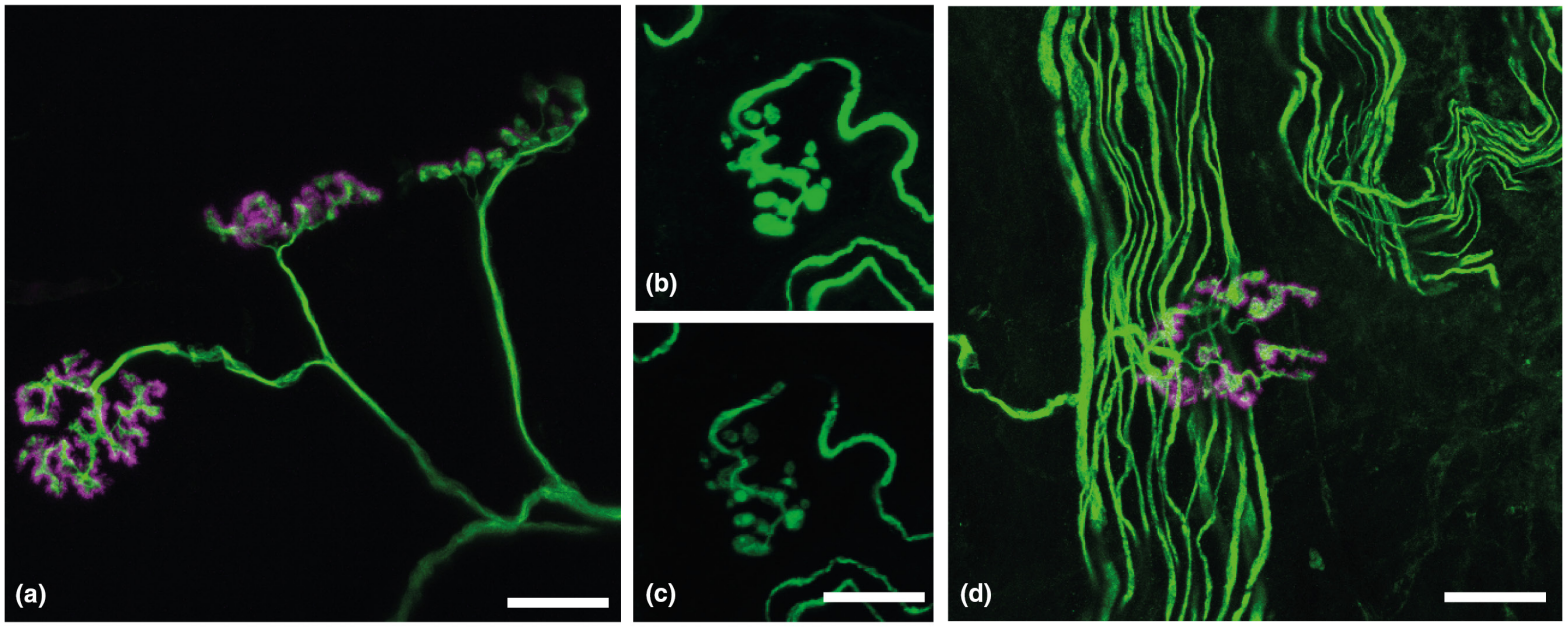
Confocal images of NMJs within equine *tibialis cranialis* muscle showing different common confocal imaging pitfalls that can generate inaccuracies in measurements of pre‐ and post‐terminal area and perimeter. Pre‐ (SV2/3A10 green) and post‐synaptic (α‐bungarotoxin, α‐BTX, magenta). (a) Progressive rotation from an *en face* view (far left), oblique (middle) and lateral view(right) rotated at a 90‐degree angle from an *en face* view; scale bar = 25 μm. (b) an overexposed distal axon and nerve terminal, with the same image (c) corrected exposure beneath, scale bar 50 μm. (d) Background staining of axons and debris can obscure NMJ morphology; scale bar = 25 μm.

NMJs were imaged on a Leica SP8 confocal microscope using established protocols reported by (Jones et al., 2016, 2017). In brief, the following settings were used: 8‐bit depth, 512 × 512 frame size, ×63magnification, ×2 zoom and 1 μm z‐stack interval with sequential image acquisition and a HC PL APO 63x/1.40 OIL CS2 oil immersion objective. On confocal microscopy, all available *en face* NMJs were imaged. See Figure 4 for common imaging pitfalls. The numbers of NMJ imaged were as follows: *N* = 5 ponies, *n* = 15 muscles, 194 *tibialis cranialis* NMJs, 169 *extensor digitorum longus* NMJs, 187 *soleus* NMJs. All imaged NMJs were analysed using ImageJ software (https://imagej.nih.gov/ij/) combined with the aNMJ‐morph plugin, a semiautomated NMJ morphology analysis workflow (Minty et al., 2020). For the purposes of analysis, ‘morphological variables’ were defined as the 19 morphological variables that are generated using aNMJ‐morph across the 15 muscles. A 20th variable of innervation‐status was visually assessed. A 21st variable of muscle fibre diameter (MFD), assessed via light microscopy, is described in section 2.10. Means ± SEM were plotted of values for all NMJs imaged within each muscle for each pony and analysed using repeated‐measures one‐way mixed model with a Bonferroni correction. An assessment, while blinded to muscle of origin, of whether nerve terminals conformed to *en grappe* or *en plaque* morphology, was completed from a Z‐stack projection of each NMJ, once NMJ‐morph analysis was completed. Assessment criteria were based on a published dataset (Hess, 1962) whereby nerve terminals were classed as either *en plaque—*a series of curled, irregular, pretzel‐like branches, or clustered as bunches of *en grappe*, spherical boutons.

### 2.9 Muscle fibre typing, immunostaining and analysis

Fresh skeletal muscle samples from muscle regions immediately adjacent to those sampled for NMJ analysis were snap‐frozen in isopentane precooled in liquid nitrogen. Thereafter, 7 μm cryosections were air‐dried onto glass slides (Superfrost plus) and stored at −80°C. Multiple antibody fluorescence labelling using a Zenon Alexafluor labelling kit was carried out, as previously described (Tulloch et al., 2011). In brief, a goat polyclonal anti‐collagen V IgG antibody (1:10 dilution) was applied for 1 h followed by PBS washes. Three separate mouse monoclonal antibodies that detect type 1 (Slow myosin heavy chain [MHC] IgG antibody, MAB1628), type 2a (Type 2a MHC IgG antibody, A4.74) and both type 2a and 2x (MHCf lgG antibody) were individually labelled respectively with Alexa fluor‐conjugated IgG1 Fab fragments designed for 3 different emitting wavelengths: 350 nm–Zenon Alexafluor 350 mouse IgG1 labelling kit; 488 nm–Zenon Alexafluor 488 mouse IgG1 labelling kit; and 594 nm–Zenon Alexafluor 594 mouse IgG1 labelling kit. The three labelled primary antibodies were mixed with a secondary Alexafluor 488 rabbit anti‐goat IgG5 secondary antibody (1:500) and applied for 1 h to the cryosection, followed by rinsing in PBS. The section was postfixed with 4% PFA in PBS for 15 min at room temperature and then washed in PBS. Sections were subsequently mounted (Vectashield mounting medium) and examined using a digital scanning fluorescence microscope (Zeiss Axioscan) with filters designed for the different emitting wavelengths. Six 20X images were captured at random per muscle, and pseudo‐coloured composites were generated for manual counting of fibres. Muscle fibre type percentages per section were calculated.

### 2.10 Muscle fibre diameter analysis

**FIGURE 5:**
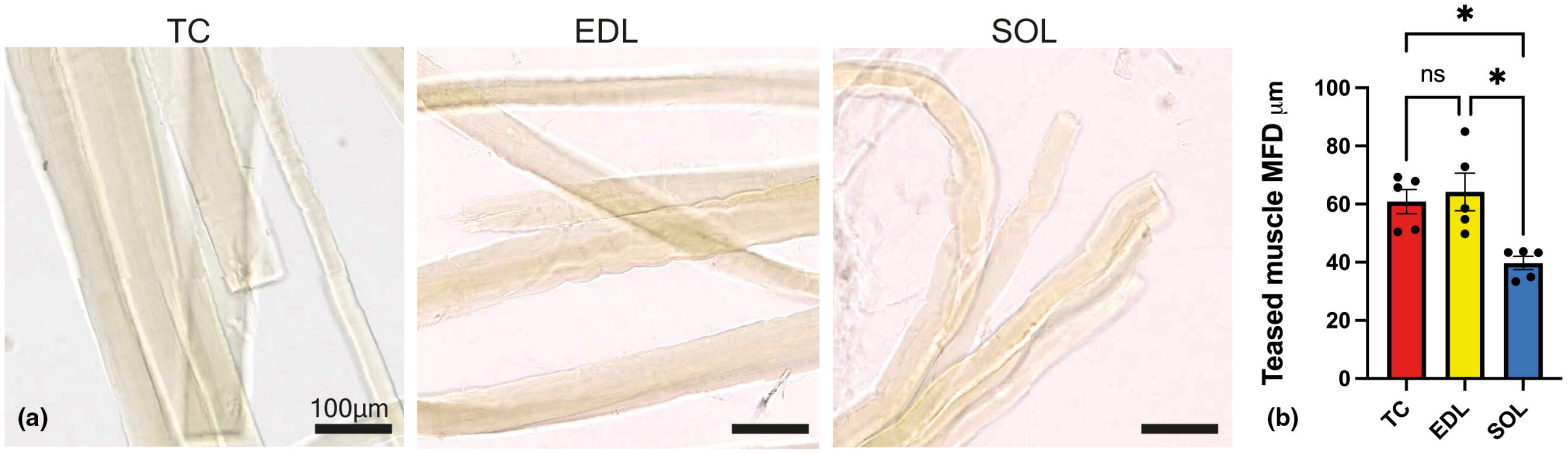
Muscle fibre diameter quantification following light microscopic imaging (following confocal imaging) of muscle fibre bundles reveals intermuscular differences. (a) Whole‐mounted fibres scanned by light microscopy using an Axioscan digital slide scanner with a 20× objective. Teased muscle fibres from the wholemount muscles showing comparatively smaller diameter *soleus* muscle fibres. Scale bars: 100 μm. (b) Mean muscle fibre diameters (MFDs) in the *tibialis cranialis* (TC) and *extensor digitorum longus* (EDL), datapoints representing mean ± SD, compared to the *soleus* (SOL). Mixed model analysis with Bonferroni's multiple comparisons test revealed a significant difference between the MFDs of the SOL, compared to the TC and EDL (*p < 0.05).

After confocal imaging of NMJs, slides with labelled NMJs in fibre bundles were scanned using an Axioscan digital slide scanner with a 20× objective (Figure 5a) using opensource software NDP.view2, v2.9.29, provided by Hamamatsu, available at www.hamamatsu.com.

Individual muscle fibres were chosen randomly, and fibre diameters were determined by taking three width measurements at their beginning, mid‐, and endpoint. Care was taken to avoid damaged or fused fibres. Forty fibres were measured per muscle per animal, frequency distributions assessed and means calculated and compared using repeated measures one‐way ANOVA mixed‐effects model analysis with Bonferroni's multiple comparisons test. Means ± SEM was plotted (*n* = 5) and individual data points were generated from each pony, i.e. the mean values from 40 fibres per muscle.

### 2.11 NMJ morphology statistical analysis

GraphPad Prism 9 (version 9.3.1) for macOS was used for all statistical analyses. The means of each morphological variable, derived from all NMJs within each pony and muscle were calculated. The mean of means was compared across each muscle using a repeated‐measures one‐way analysis of variance (ANOVA), mixed‐effects model, with pairwise comparisons compared with Bonferroni's multiple comparisons test. Correlation against body weight, muscle fibre type and diameter were assessed using Pearson's product–moment correlation (*p* < 0.05).

### 2.12 Principal component analysis

Principal component analysis (PCA) was used for dimensionality reduction of the large dataset of pony NMJ morphological variables, condensing the existing variables generated by aNMJ‐morph into new principal components (PCs) created through linear combinations of the original dataset and plotted as PC scores. The variance between each PC is maximised, thus revealing additional information about the dataset by illustrating which variables contribute most. Loading analysis revealed how strongly each morphological value contributed to each PC. PCA plots and loading analysis were created using GraphPad Prism 9 (version 9.3.1). Using the PC scores plot of all combined averaged data, the percentage of overlapping data points for individual NMJs per muscle were calculated.

## 3 RESULTS

### 3.1 NMJs in the equine *soleus* muscle are morphologically distinct from those in the *extensor digitorum longus* and *tibialis cranialis* muscle

**FIGURE 6:**
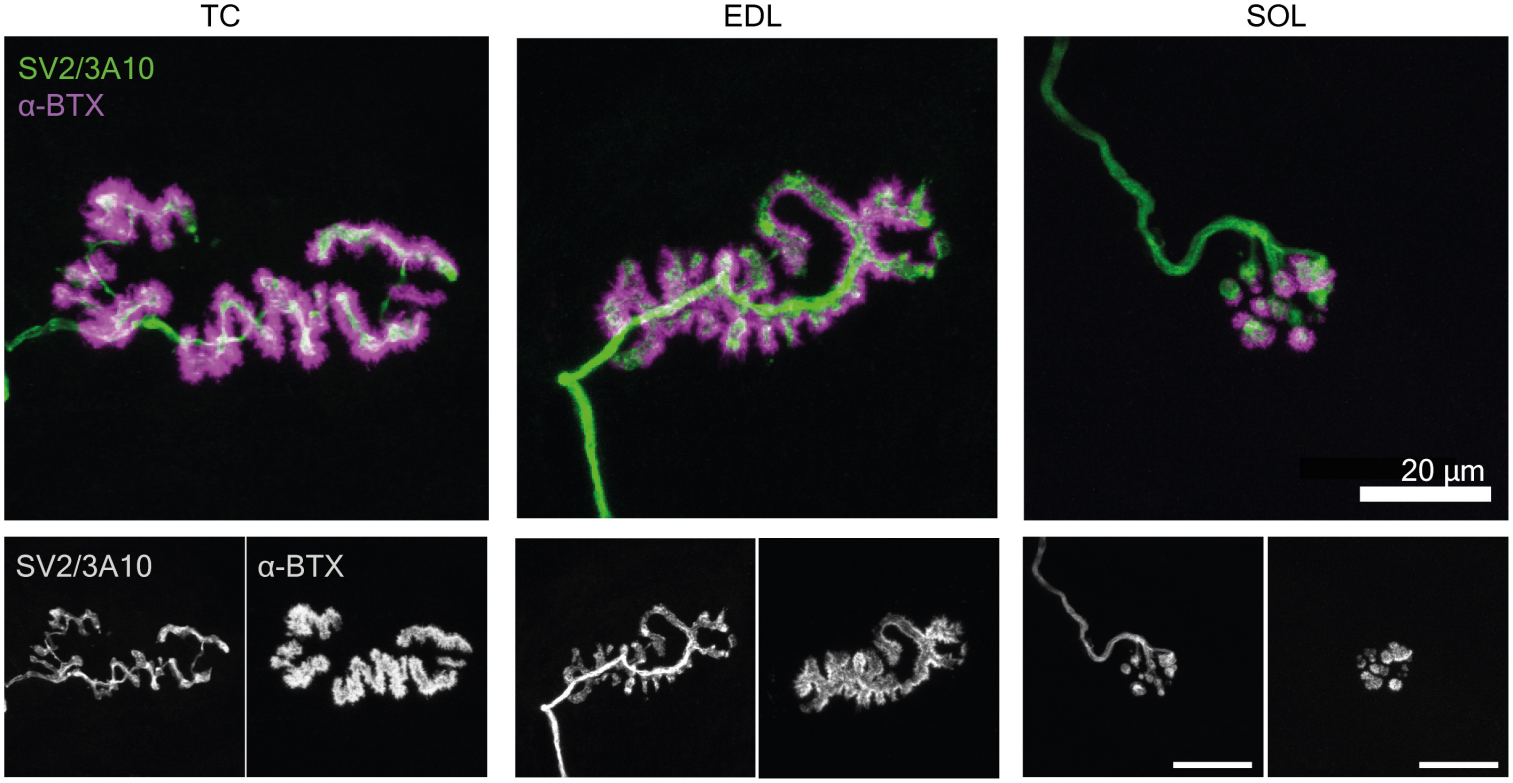
Nerve terminals have a distinct *en plaque* morphology in tibialis cranialis and *extensor digitorum longus* muscles, in contrast, the *soleus* muscle has *en grappe* terminals. Presynaptic terminal (SV2/3A10 in green) and endplate (α‐bungarotoxin, α‐BTX, in magenta). (a) Representative fluorescence micrographs of *tibialis cranialis* (TC), *extensor digitorum longus* (EDL) and *soleus* (SOL) muscle NMJs showing *en plaque* (TC and EDL) and *en grappe* (SOL) nerve terminal morphology.

**FIGURE 7:**
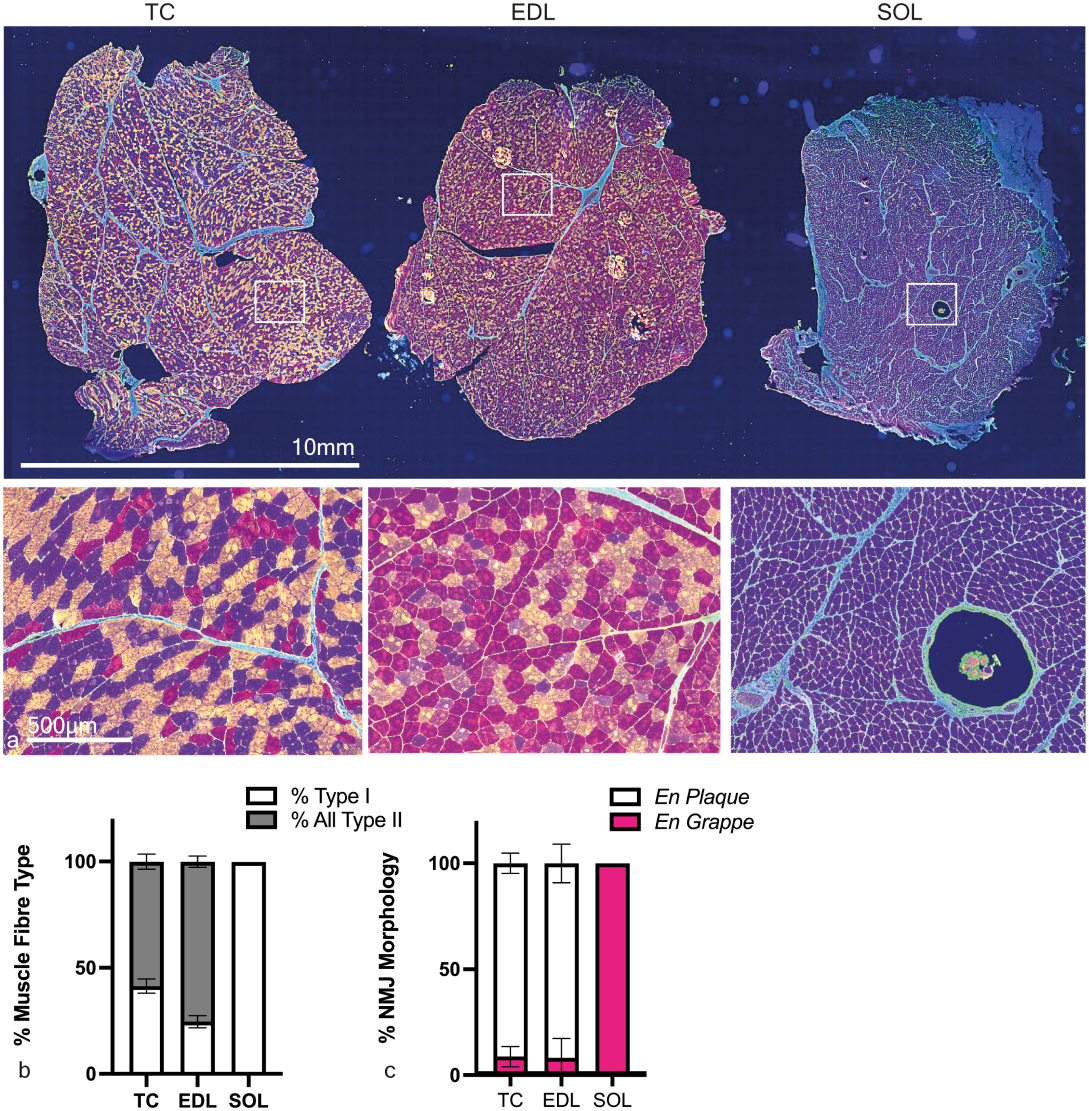
Muscle fibre type distributions and NMJ morphologies per muscle revealed with immunolabelling. (a) representative fluorescence micrographs of *tibialis cranialis* (TC), *extensor digitorum longus* (EDL) and *soleus* (SOL) muscle. The boxed areas in the upper row of images are represented at higher magnification in the lower row. Pseudocoloured: Blue = type 1 fibres, green = collagen, red = type 2× fibres, yellow = type 2a, orange = type 2ax hybrid fibres. A muscle spindle is present in the soleus muscle, bottom right. (b) Means ± SD percentage muscle fibres per muscle per pony, from six 20X images, captured at random, per muscle. *N* = 5 ponies, *n* = 15 muscles; *tibialis cranialis* and *extensor digitorum longus* muscle had a majority of type 2 fibres whereas almost all *soleus* muscle fibres were type 1. (c) Means ± SD percentage NMJ morphologies, assessed while blinded, from maximum z‐stack projections (TC NMJs = 194, EDL NMJs = 168, SOL NMJs = 187); where *tibialis cranialis* and *extensor digitorum longus* muscle had a majority of *en plaque* NMJs, *soleus* showed exclusively *en grappe* NMJs.

AChE staining identified the typical gross distribution of endplate‐rich areas as a series of overlapping bands on the muscle surface (Figure 1), repeating along the entire length of the muscle, indicating target regions for muscle fibre teasing in subsequent samples. Following immunolabelling of teased fibres, the equine NMJ revealed motor nerve terminals and endplates that were closely morphologically aligned across the three muscles: the distal nerve terminals were singly innervated, branched and varicose, and ACh receptors were highly concentrated at endplates, with striations revealing the orientations of postsynaptic folds (Figure 6). Intermuscular differences in distal nerve terminal morphologies were revealed, showing *tibialis cranialis* (TC, NMJs = 200) and *extensor digitorum longus* (EDL, NMJs = 168) muscles with, respectively, 91.2% [±4.7] and 91.7 [±9.1] (mean [SD]) curled branch or irregular pretzel‐like *en plaque* terminals, while *soleus* (SOL, NMJs = 187) nerve terminals appeared exclusively in grape‐like or *en grappe* clustered spherical boutons (Figure 7c). Mean percentages of muscle fibre types (Figure 7b), calculated per muscle per pony showed 58.5% (±14.4) and 75% (±9.6) type 2 muscle fibres within the TC and EDL, respectively. Whereas the SOL contained almost exclusively type 1 fibres (>99%), except for occasional type 2 labelled muscle spindles (Figure 7a). Type IIa fibres represented 37.3% (±7.9) and 43.5% (±14.1) of the TC and EDL type II fibre total, respectively, with 21.1% (±7.9) and 32.2% (±20.81) being IIx fibres respectively.

### 3.2 Modest intermuscular NMJ morphological variability revealed by aNMJ‐morph analysis

**FIGURE 8:**
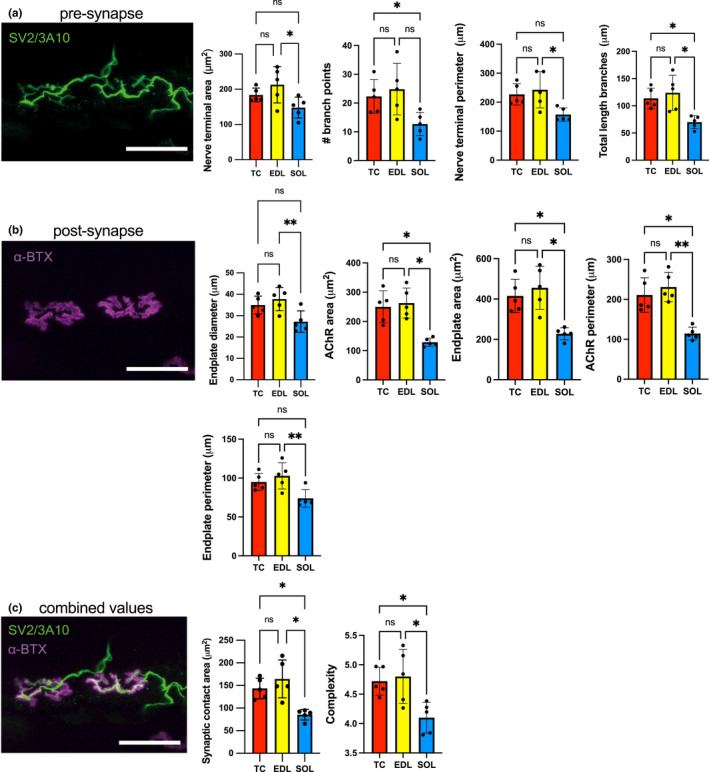
aNMJ‐morph reveals inter‐muscular morphological variability. (a) Three different muscles were dissected from five ponies and immunohistochemically processed to visualise and assess pre‐ (SV2/3A10, green) and post‐synaptic (α‐bungarotoxin, α‐BTX, magenta) NMJ morphology. The image is of a *tibialis cranialis* muscle NMJ. Scale bars: 50 μm. The muscle colour scheme: yellow, *extensor digitorum longus* (EDL); red, *tibialis cranialis* (TC) and blue, *soleus* (SOL). Means ± SEM per muscle of each NMJ morphological variable were plotted in columns against the means of the two other muscles (repeated measures) from each pony. Of 21 morphological variables analysed using repeated‐measures one‐way mixed model analysis, moderately significant differences were limited to EDL and SOL muscles post‐synaptically only. Significant differences were noted in pre‐synaptic (a), post‐synaptic (b) and overlapping/combined (c) values between muscles *p < 0.05, **p < 0.01, ***p < 0.001.

From a comparison of 21 variables (19 morphological variables generated through aNMJ‐morph and the 2 associated variables of axonal input and MFD) and across the three muscles (see Table 5), significant differences (**p < 0.01) were detected in the core post‐synaptic variables of endplate diameter and perimeter and AChR perimeter, occurring between the EDL and SOL muscles only (Figure 8b). Of the rest, significant (*p < 0.05) differences were noted between either the EDL and the SOL and/or TC and the SOL for pre‐synaptic and post‐synaptic variables and ‘combined’ values derived from combinations of pre‐ and postsynaptic measurements (Figure 8a–c, respectively). As a direct measure of alignment, the lowest percentage overlap between endplate and nerve terminal in the ponies was 58.49% for the TC, and the highest was 67.33% for the SOL; no significant difference was noted between these or the remaining values (see Table 5).

**TABLE 5:** Average morphological data for pony pelvic limb muscles generated in aNMJ‐morph

	TC *N* = 5, *n* = 5200 NMJs	EDL *N* = 5, *n* = 5 68 NMJs	SOL *N* = 5, *n* = 5187 NMJs
Core variables			
Pre‐synaptic			
(1) Nerve terminal area (μm^2^)	183.7 ± 4.62	215.3 ± 6.46*	150.9 ± 4.65
(2) Nerve terminal perimeter (μm)	227.1 ± 5.31	244.5 ± 7.54*	158.2 ± 4.47
(3) Number of terminal branches	29.43 ± 1.10	31.57 ± 1.43	21.73 ± 0.89
(4) Number of branch points	22.57 ± 1.05*	24.46 ± 1.08	12.67 ± 0.64
(5) Total length of branches (μm)	114.1 ± 3.06*	124.7 ± 4.03*	70.06 ± 2.24
Post‐synaptic			
(6) AChR area (μm^2^)	248.6 ± 6.39*	270.1 ± 7.34*	131.5 ± 3.94
(7) AChR perimeter (μm)	211.2 ± 5.40*	219.0 ± 6.9**	114.9 ± 3.32
(8) Endplate area (μm^2^)	414.9 ± 11.61*	460.4 ± 15.92*	232.0 ± 7.84
(9) Endplate perimeter (μm)	95.08 ± 1.51	103.0 ± 2.33**	74.77 ± 1.81
(10) Endplate diameter (μm)	35.00 ± 0.64	37.63 ± 0.8**	27.44 ± 0.71
(11) Number of AChR clusters	2.49 ± 0.12	2.47 ± 0.16	3.01 ± 0.22
Derived variables			
Pre‐synaptic			
(12) Average length of branches (μm)	4.56 ± 0.18	4.73 ± 0.18	3.72 ± 0.13
(13) Complexity	4.72 ± 0.03*	4.77 ± 0.05*	4.10 ± 0.04
Post‐synaptic			
(14) Average area of AChR clusters (μm^2^)	151.2 ± 7.87	160.5 ± 8.37	62.96 ± 4.89
(15) Fragmentation	0.41 ± 0.02	0.39 ± 0.02	0.54 ± 0.03
(16) Compactness (%)	61.11 ± 0.6	61.58 ± 0.79	58.80 ± 0.80
(17) Overlap (%)	58.49 ± 0.77	62.97 ± 0.84	67.33 ± 0.75
(18) Area of synaptic contact (μm^2^)	142.7 ± 3.69*	168.9 ± 4.93*	88.41 ± 2.56
Associated nerve & muscle variables			
(19) Axon diameter (μm)	1.582 ± 0.05	1.65 ± 0.06	2.104 ± 0.05
(20) Muscle fibre diameter	60.90 ± 1.24*	64.19 ± 1.49*	39.72 ± 0.75
(21) Number of axonal inputs	1 ± 0.0	1 ± 0.0	1 ± 0.0

*Note*: Means ± SEM of each morphological variable per muscle; core variables (1–11), derived variables (12–19) and associated nerve and muscle variables (19–21). *N* = animals, *n* = muscles, and total NMJs analysed are listed: *Tibialis cranialis* (TC, 200 NMJs), *extensor digitorum longus* (EDL, 168 NMJs) and *soleus* (SOL, 187 NMJs), corresponding to the data shown in Figures 8 and 9. Statistical difference between the *soleus* and the other two muscles were compared using repeated‐measures one‐way mixed model analysis; *p < 0.05; **p < 0.01; ***p < 0.001; no asterisk = non‐significant result.

Mean muscle fibre diameters (MFDs) in TC and EDL fibres, were 60.9 μm ± 4.2 and 64.2 μm ± 6.5 respectively (mean ± SEM), compared to 39.7 μm ± 2.3 in the SOL (Figure 5b). Mixed model analysis with Bonferroni's multiple comparisons test revealed a significant difference between the MFDs of the SOL, compared to the TC and EDL (*p < 0.05). MFD values correlated with synaptic contact area (*r* = 0.79, *p* = 0.0004) and with AChR area (*r* = 0.772, *p* = 0.0007). No significant correlation was noted between aNMJ‐morph–derived variables and body weight or muscle fibre type (data not shown).

### 3.3 Principal component analysis separates morphologically distinct NMJs into muscle‐associated groups

**FIGURE 9:**
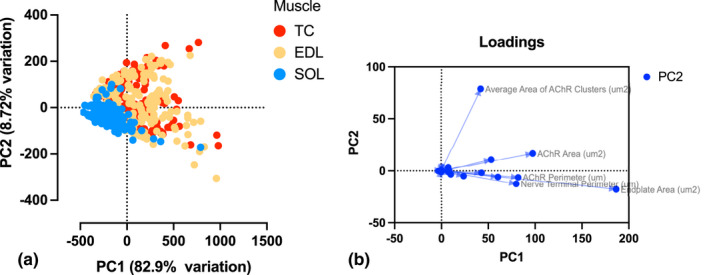
Principal component analysis separates morphologically distinct NMJs. Two‐dimensional PCA representations of the NMJs as defined by the 20 morphological variables (19 variables describing NMJ morphology and muscle fibre diameter). (a) PC scores of the complete set of NMJs, colour‐coded by muscle type. PC1 on the x‐axis accounted for 82.49% of the variation in the dataset, separating most of the SOL NMJs from those of the TC and EDL. PC2 on the y‐axis explained 8.72% of the variation within the dataset. Both TC (35%) and EDL (17.5%) NMJ datapoints overlap with those of the soleus NMJs. (b) PCA loadings map from panel (a), illustrating the endplate area contribution to PC1 and the average area of AChR cluster contribution to PC2.

The complete dataset composed of 20 variables (19 morphological variables generated through aNMJ‐morph and MFD, less the number of axonal inputs) was condensed into linear combinations and plotted along the first two most important principal components (PCs) (Figure 9a). PC1 accounted for 82.9% of the variation in the dataset, clearly separating most of the SOL NMJs from those of the TC and EDL. PC2 explained 8.72% of the variation within the dataset, yet it also separated SOL NMJs from the other two muscles. An even distribution of TC and EDL datapoints, plotted across PC 1 and 2, indicated a similarity between NMJ variables for those muscles, yet only 35% of TC and 17.5% of EDL datapoints overlapped with those of the SOL. Loadings plot analysis of the 20 variables showed that endplate area contributed most to PC1, with nerve terminal perimeter and AChR area and perimeter also contributing. Average area of AChR clusters contributed most to PC2 (Figure 9b)

## 4 DISCUSSION

This paper presents NMJ morphology data from the equine pelvic limb and addresses the main challenges of working with large mammalian muscles: (1) to detect gross endplate distribution; (2) to reduce fixation‐ and muscle fibre teasing‐associated artefacts; and (3) to obtain sufficient antibody penetration during immunolabelling, prior to high‐resolution confocal imaging. We reveal the organisation of equine NMJ morphology to be similar to that of other vertebrates and report NMJ morphological variability between equine pelvic limb muscles with different muscle fibre type proportions.

Similar to many long muscles in humans and other vertebrates, multiple end‐plate bands were evident in the equine EDL, TC and SOL, suggesting that most of the constituent fibres do not run from origin to insertion, given that NMJs are typically located in the middle of the muscle fibre (Bianchi & Bianchi, 2018; Patton & Burgess, 2005). This paper's muscle fibre teasing method, adapted from studies in smaller mammals and humans (Boehm, Alhindi, et al., 2020), successfully yielded quantifiable NMJs for analysis with minimal staining artefacts, indicating its cross‐species compatibility.

The equine NMJ has presynaptic vesicle clusters within terminal boutons that are well aligned to postsynaptic ACh receptors, regardless of the differences in nerve terminal shape between these muscles. Thus, highlighting a stereotypical organisation that also occurs across other vertebrate species (Boehm, Alhindi, et al., 2020;Patton & Burgess, 2005; Petralia et al., 2017). *Soleus* muscle nerve terminals had exclusively an *en grappe* morphology, in marked contrast to the TC and EDL, which were mostly *en plaque*. Distinct nerve terminal morphologies have been reported for different muscles across vertebrates—in general, smaller single boutons or *en grappe*–like terminals are noted on muscle fibres that undergo slow and sustained (tonic) contraction, whereas longer branched, *en plaque*–like nerve terminals are seen on fast twitch fibres (Patton & Burgess, 2005; Petralia et al., 2017). Exceptions to this generalisation exist, highlighted by the discrepancy between fibre type percentage and NMJ morphology in the TC and EDL muscles (Figure 7), indicating that not all type I fibres in these muscles have an *en grappe* shape.

NMJ morphological heterogeneity has been reported in select pelvic limb muscles (limited by availability of comparative human NMJ data (Jones et al., 2017)) within and across 6 other mammalian species in a recent NMJ study by Boehm, Alhindi, et al. (2020). Similar to their within‐species findings, most core pre‐ and postsynaptic variables in the ponies showed either only limited or modest intermuscular differences—the most significant of those, backed by mixed model and principal component analysis, occurred post‐synaptically between the SOL and the EDL, the smallest and largest pony NMJs respectively. Boehm et al revealed that the mouse and human soleus contains the largest relative NMJs, whereas sheep and pig contain the smallest; a comparison of representative pony EDL NMJs (the only muscle present in all seven species) shows that, in general, the pony has a larger terminal axon than that of the human NMJ, yet its endplates are similarly fragmented. aNMJ‐morph‐generated variables did not significantly correlate with body weight or muscle fibre diameter in the pony, similar to the other mammalian species studied and to previous reports in humans (Jones et al., 2017)—the common factor that governs NMJ morphological variation across species is not thus far evident, highlighting a translational gap in the understanding of NMJ form, which perhaps can be explained by species‐specific differences in biomechanics‐driven motor unit function.

Differences in NMJ shape and postsynaptic morphology between the SOL and EDL are particularly mirrored in each muscle's size and function. Equine soleus muscles are relatively small, representing approximately 0.01% of total equine pelvic limb muscle mass–a figure derived from data across two studies containing a combined 20 mix‐breed and Thoroughbred horses (Meyers & Hermanson, 2006; Payne et al., 2005). In contrast, TC and EDL muscles represent 0.6% and 0.93% respectively, and the *gluteus medius* at 17.33% is the largest relative muscle. Large differences also exist between the MFDs of the SOL compared to the TC and EDL—perhaps explained by muscle fibre type differences. The presumed fibre types reported for TC (Valberg et al., 2015) and EDL (Kawai et al., 2009) (see Table 2) are close to those noted in these ponies and as reported previously (Meyers & Hermanson, 2006), the pony SOL sections contained almost 100% slow‐twitch fibres. Across mammals, the *soleus* muscle typically contains a majority of slow‐twitch fibres (Schiaffino & Reggiani, 2011), with the predominant equine type I skew likely reflecting its purported minor role in hock extension (Payne et al., 2005), but also crucially, a postural or tonic contractile one (Meyers & Hermanson, 2006). This is in contrast to the TC and EDL's function in hock flexion and, in the latter, digit extension and locomotion. Our findings thus suggest a relationship between pony muscle fibre type/function and NMJ morphology that is, smaller diameter SOL fibres accompanied by smaller *en grappe* NMJs—perhaps driven by species‐specific functional demands for weakly sustained synaptic transmission/contraction, compared to the longer branched EDL and TC NMJs aiding more vigorous contraction. A similar relationship between MFD and NMJ size has been shown in a select range of muscles within multiple species (Ogata, 1988) and rodent diaphragm (Prakash et al., 1996); yet other rodent distal limb studies contrast this with larger NMJs observed in muscles with lower fast‐twitch fibre percentages (Mech et al., 2020).Recent comparative work highlights a clear disconnect between NMJ morphology and muscle fibre size/identity within select human and rodent pelvic limb muscles (Jones et al., 2017) and within the same muscle set across four other species (Boehm, Alhindi, et al., 2020). Given the interspecies fibre type variation reported within the SOL, with smaller species containing markedly more type II muscle fibres (Schiaffino & Reggiani, 2011), perhaps our equine NMJ findings highlight the difficulty in making translational cross‐species comparisons, even within the same muscle, as motor neurons have evolved within each species to meet their specific biomechanical and functional demands at the nerve terminal. These likely contribute to cross‐species variations in NMJ morphology, with some recent evidence to support this (Jones et al., 2017).

Caveats in our data include sample location, with EDL and TC samples, in particular, reflecting a small percentage of the overall muscle mass and an inability to directly correlate the MFD and fibre type to each respective NMJ. Differences in anatomic sample location and methodology likely also underpin some of the variations noted both within‐ and between species. An area for further cross‐species study is the development of a method to co‐label/identify NMJ and muscle fibres for concurrent morphological and volumetric analysis.

## 5 CONCLUSION

Despite the technical difficulties inherent in large mammalian muscle experiments, the techniques outlined show that equine muscles can have their NMJ morphology visualised using immunofluorescence confocal microscopy. We have used pony tissue to demonstrate the cross‐species translational potential of muscle preparation for highlighting NMJ phenotypes in large mammals and describe the detailed morphology of equine NMJs.

## AUTHOR CONTRIBUTIONS

SDC, JDP, IB, RAJ, THG and RJP conceived and designed the study. IB and SDC performed experiments and analysed data. SDC, IB, JDP, RAJ, THG and RJP wrote the manuscript. All authors edited and approved the manuscript and have no conflict of interest to declare.

## FUNDING INFORMATION

Horserace Betting Levy Board (HBLB) Grant & Anatomical Society Prize PhD Studentship.

### OPEN RESEARCH BADGES

This article has earned an Open Data badge for making publicly available the digitally‐shareable data necessary to reproduce the reported results. The data is available at https://doi.org/10.5281/zenodo.6519637.

## Supporting information


Appendix S1

## Data Availability

Not applicable.
